# *Pseudomonas aeruginosa* Enolase Influences Bacterial Tolerance to Oxidative Stresses and Virulence

**DOI:** 10.3389/fmicb.2016.01999

**Published:** 2016-12-15

**Authors:** Yuding Weng, Fei Chen, Yiwei Liu, Qiang Zhao, Ronghao Chen, Xiaolei Pan, Chang Liu, Zhihui Cheng, Shouguang Jin, Yongxin Jin, Weihui Wu

**Affiliations:** ^1^State Key Laboratory of Medicinal Chemical Biology, Key Laboratory of Molecular Microbiology and Technology of the Ministry of Education, Department of Microbiology, College of Life Sciences, Nankai UniversityTianjin, China; ^2^Department of Molecular Genetics and Microbiology, College of Medicine, University of Florida, GainesvilleFL, USA

**Keywords:** *Pseudomonas aeruginosa*, enolase, oxidative stress response, bacterial virulence, gene regulation

## Abstract

*Pseudomonas aeruginosa* is a Gram negative opportunistic pathogenic bacterium, which causes acute and chronic infections. Upon entering the host, bacteria alter global gene expression to adapt to host environment and avoid clearance by the host. Enolase is a glycolytic enzyme involved in carbon metabolism. It is also a component of RNA degradosome, which is involved in RNA processing and gene regulation. Here, we report that enolase is required for the virulence of *P. aeruginosa* in a murine acute pneumonia model. Mutation of enolase coding gene (*eno*) increased bacterial susceptibility to neutrophil mediated killing, which is due to reduced tolerance to oxidative stress. Catalases and alkyl hydroperoxide reductases play a major role in protecting the cell from oxidative damages. In the *eno* mutant, the expression levels of catalases (KatA and KatB) were similar as those in the wild type strain in the presence of H_2_O_2_, however, the expression levels of alkyl hydroperoxide reductases (AhpB and AhpC) were significantly reduced. Overexpression of *ahpB* but not *ahpC* in the *eno* mutant fully restored the bacterial resistance to H_2_O_2_ as well as neutrophil mediated killing, and partially restored bacterial virulence in the murine acute pneumonia model. Therefore, we have identified a novel role of enolase in the virulence of *P. aeruginosa*.

## Introduction

*Pseudomonas aeruginosa* is a ubiquitous Gram negative bacterium. It is a major cause of nosocomial infections, including severe burn infections, sepsis, urinary tract infection, and pneumonia. Of note, *P. aeruginosa* is the leading cause of chronic lung infection in cystic fibrosis (CF) patients and ventilator-associated pneumonia (VAP; Diaz et al., 2005; Mcbride, 2005).

During infection, *P. aeruginosa* orchestrates expression of multiple virulence factors to counteract host immune clearance and increase tolerance to antibiotics (Rahme et al., 2000; Poole, 2011; Balasubramanian et al., 2012; Morita et al., 2014). In a murine acute pneumonia model, it has been demonstrated that neutrophils are rapidly recruited to the lung in response to invading bacteria (Shaver and Hauser, 2004). Neutrophils are phagocytes that kill bacteria by generation of reactive oxygen species (ROS), phagocytosis, and degranulation (Ziltener et al., 2016).

Among the virulence factors of *P. aeruginosa*, the type III secretion system (T3SS) plays an essential role in killing phagocytes or inhibiting phagocytosis (Brannon et al., 2009; Plano and Schesser, 2013). The T3SS is a needle like structure conserved in various Gram negative animal and plant pathogenic bacteria, through which effector proteins are directly injected into host cell cytosol, altering cell signaling, or killing host cells (Luo and Jin, 2008; Bleves et al., 2010; Pha and Navarro, 2016). Four effector proteins, namely ExoU, ExoS, ExoT, and ExoY have been identified in *P. aeruginosa* (Hornef et al., 2000). Most clinic isolates express three of the four effectors, including ExoT, ExoY and either ExoU, or ExoS (Feltman et al., 2001; Shaver and Hauser, 2004). Injection of the ExoS or ExoU into phagocytes is critical for the pathogenesis of *P. aeruginosa* in a murine acute pneumonia model (Shaver and Hauser, 2004).

Meanwhile, *P. aeruginosa* expresses catalases (KatA and KatB) and alkyl hydroperoxide reductases (AhpB and AhpC) to defend against host produced ROS (Lee et al., 2005). Expression of these antioxidant genes is activated by a transcriptional regulator OxyR in response to oxidative stresses, such as H_2_O_2_ (Ochsner et al., 2000). OxyR contains two conserved cysteine residues, oxidation of which results in formation of an intramolecular disulfide bond, promoting the binding between OxyR and target promoters (Jo et al., 2015).

Multiple regulatory proteins and RNAs are involved in the regulation of virulence factors. The bacterial RNA degradosome, which is composed of polynucleotide phosphorylase (PNPase), enolase, RNA helicase (RhlB) and ribonuclease E (RNase E), plays an important role in RNA processing and gene regulation (Favaro and Deho, 2003; Burger et al., 2011; Matos et al., 2011; Saramago et al., 2014). Previously, we demonstrated that PNPase is required for the expression of T3SS genes and pathogenesis of *P. aeruginosa* in a murine acute pneumonia model (Chen et al., 2016). These results prompted us to explore the functions of other RNA degradosome components in bacterial pathogenesis. Enolase is another key component of RNA degradosome. It is highly conserved in bacteria (Canback et al., 2002). Studies on the *Escherichia coli* RNA degradosome revealed that enolase binds to a small region in the degradosome-scaffolding domain of RNase E (Chandran and Luisi, 2006). A crystal structure analysis suggested that enolase may facilitate the organization of a RNA-binding motif in RNase E (Nurmohamed et al., 2010). In *E. coli*, it has been shown that enolase, but not PNPase or RhlB, is required for the RNase E mediated degradation of the glucose transporter PtsG mRNA in response to metabolic stress (Morita et al., 2004). The function of enolase in *P. aeruginosa* is not well known. Here in this study, we found that enolase is essential for the virulence of *P. aeruginosa* in a murine acute pneumonia model. Instead of affecting T3SS gene expression, enolase is required for bacterial oxidative stress response. Thus, our results revealed a novel role of enolase in bacterial pathogenesis.

## Materials and Methods

### Bacterial Strains, Plasmids, and Growth Conditions

Strains and plasmid used in this study are listed in **Table 1**. For the construction of an *eno* deletion mutant, a 934-bp upstream fragment and a 1207-bp downstream fragment of the *eno* conding region were amplified by PCR with PAK chromosome as the template and primers shown in Supplementary Table S1. The fragments were cloned into the plasmid pEX18TC (Hoang et al., 1998). Deletion of the *eno* gene in *P. aeruginosa* was performed as previously described (Hoang et al., 1998). For the complementation of *eno*, the *eno* gene and its native promoter were amplified with primers shown in Supplementary Table S1. The fragments were ligated into pUC18T-mini-Tn7T-Gm. The plasmid was transferred into the *eno* mutant strain along with the helper plasmid pTNS3 by electroporation as previously described (Choi and Schweizer, 2006). The *ahpB* and *ahpC* coding regions were amplified with primers shown in Supplementary Table S1 and ligated into pUCP20, respectively. The plasmid was transferred into the *eno* mutant by electroporation. To construct the *ahpB-* and *ahpC-lacZ* transcriptional fusions, the promoter regions of *ahpB* and *ahpC* were amplified with primers shown in Supplementary Table S1 by PCR. The fragments were ligated into the vector pDN19lacZΩ (Li et al., 2013).

**Table 1:** Strains and plasmids.

Strain or plasmid	Relevant characteristics or function	Reference or origin
***E. coli* strains**		
DH5α	F^-^, ϕ80d*lac*ΔM15,Δ(*lacZYA-argF*)*U169, deoR, recA1, endA1, hsdR17(r_k__-_,m _k_^+^), phoA, supE44, λ _-_, thi1, gyrA96, relA1*	TransGen


S17-1	*Thi pro hsdR recA Tra^+^*	Simon et al., 1983


***P. aeruginosa***		


PAK	Wild-type *P. aeruginosa* strain	David Bradley


Δ*eno*	PAK with deletion of *eno* gene	This study


Δ*eno*/*eno*	Δ*eno* complemented by a wild type *eno* gene driven by its native promoter	This study


PAK/pUCP20	Wild-type PAK with plasmid pUCP20	This study
Δ*eno*/pUCP20	Δ*eno* mutant strain with plasmid pUCP20	This study
Δ*eno*/*ahpB*-pUCP20	Overexpression of *ahpB* in the Δ*eno* mutant	This study
Δ*eno*/*ahpC*-pUCP20	Overexpression of *ahpC* in the Δ*eno* mutant	This study
Δ*eno*/*ahpBC*-pUCP20	Overexpression of *ahpB* and *ahpC* in the Δ*eno* mutant	This study
**Plasmids**		
pUC18T-mini-Tn7T-Gm	For gene insertion in chromosome; Gm^r^	Choi and Schweizer, 2006
pTNS3	Helper plasmid	Choi and Schweizer, 2006
pEX18Tc	Broad-host-range gene replacement vector	Hoang et al., 1998
*eno*-pUCT-mini-Tn7T-Gm	Plasmid with an *eno* gene driven by its native promoter for chromosomal insertion	This study
*ahpB*-pUCP20	Overexpression of *ahpB*	This study
*ahpC*-pUCP20	Overexpression of *ahpC*	This study
*ahpBC*-pUCP20	Overexpression of *ahpB* and *ahpC*	This study
pDN19lacZΩ	Promoterless *lacZ* fusion vector; Sp^r^Sm^r^Tc^r^	Li et al., 2013
P*ahpB-*pDN19lacZΩ	*ahpB* promoter of PAK fused to promoterless *lacZ* on pDN19lacZΩ; Sp^r^Sm^r^Tc^r^	This study
P*ahpC*-pDN19lacZΩ	*ahpC* promoter of PAK fused to promoterless *lacZ* on pDN19lacZΩ; Sp^r^Sm^r^Tc^r^	This study


All bacterial strains were cultured in Luria broth (LB, 1% Bacto-tryptone, 0.5% yeast extract, 1% NaCl; Oxoid Ltd, USA) at 37°C. Antibiotics were used at the following concentrations: for *E. coli*, kanamycin 50 μg/ml, gentamicin 15 μg/ml; for *P. aeruginosa*, carbenicillin 150 μg/ml, gentamicin 50 μg/ml, tetracycline 50 μg/ml. All antibiotics are from BBI Life Science, Shanghai, China.

### β-Galactosidase Assay

β-Galactosidase assay was performed as previously described (Miller, 1972) with minor modifications. Briefly, bacteria were cultured overnight and diluted 1:100 in fresh LB medium and grown at 37°C with agitation. When the optical density at 600 nm (OD_600_) reached 2.0, 0.5 ml bacteria were collected by centrifugation and resuspended in 1.5 ml Z buffer (60 mM Na_2_HPO_4_, 60 mM NaH_2_PO_4_, 10 mM KCl, 1 mM MgSO_4_, 50 mM β-mercaptoethanol, pH 7.0; BBI Life Science, Shanghai, China). One milliliter of the suspension was allocated for OD_600_ measurement. The other 0.5 ml suspension was added with 10 μl chloroform (BBI Life Science, Shanghai, China) and 10 μl 0.1% SDS (BBI Life Science, Shanghai, China), followed by vortex for 10 s. Then 100 μl ONPG (40 mg/ml; Sigma, USA) was added to the mixture and incubated at 37°C. The reaction was stopped by addition of 0.5 ml 1M Na_2_CO_3_. The time was recorded and OD_420_ was measured. β-Galactosidase activity (Miller units) was calculated as (1000 × OD_420_)/(T × V × OD_600_). T, reaction time (minute); V, bacteria volume (ml).

### Murine Acute Pneumonia Model

Infection of mouse was performed as previously described (Sun et al., 2014). Briefly, overnight bacterial culture was diluted 1:100 in fresh LB medium and grown at 37°C with agitation. When the optical density at 600 nm (OD_600_) reached 1.0, bacteria were collected and resuspended in phosphate-buffered saline (PBS) at a concentration of 1 × 10^9^ CFU/ml. Six to eight weeks old female BALB/c mice (Vital River, Beijing, China) were anesthetized by the injection of 100 μl 7.5% chloral hydrate (Sigma, USA) intraperitoneally. Twenty microliter bacterial suspension was then inoculated intranasally to each mouse, resulting in 2 × 10^7^ CFU bacteria per mouse. Twelve hours post-infection (hpi), the mice were sacrificed and lungs were isolated and homogenized in 1% proteose peptone (Sigma, USA), followed by determination of bacterial loads by serial dilution and plating. In the mortality assay, each mouse was infected with 4 × 10^7^ CFU bacteria, and monitored for 6 days. The statistical analysis was performed with the Prism software (Version 6, Graphpad Software, La jolla, USA).

### RNA Extraction and Real Time PCR (qRT-PCR)

Total RNA was isolated with the RNA prep Pure cell/Bacteria Kit (Tiangen Biotec, Beijing, China). Random primers and the Prime Script Reverse Transcriptase (Takara, Dalian, China) were used to synthesize cDNA. The cDNA was used as the template to detect the relative mRNA levels of indicated genes with specific primers and Fast Start Essential DNA Green Master (Roche, Switzerland). Gene PA1805 was used as the internal control (Son et al., 2007).

### Histology

Twelve hours after infection with indicated *P. aeruginosa* strains, lungs of the mice were removed and fixed with 10% paraformaldehyde (Sigma, USA), then dehydrated with ethanol (Tian Jin chemical reagent company, Tianjin, China), and embedded in paraffin (BBI Life Science, Shanghai, China). The tissue sections were cut into slices and stained with hematoxylin and eosin (BBI Life Science, Shanghai, China). Images were taken with an Olympus microscope (Version IX71, Tokyo, Japan).

### Cytotoxicity Assay

Bacterial cytotoxicity was determined by the lactate dehydrogenase (LDH) release assay. Briefly, HeLa cells (ATCC, USA) were cultured in Dulbecco’s modified Eagle medium (DMEM, Hyclone, USA) with and 2% (vol/vol) heat-inactivated fetal bovine serum (hiFBS, Gibco, Australia) at 37°C with 5% CO_2_. Eighteen hours before infection, 1.2 × 10^5^ HeLa cells were seeded into each well of a 24-well plate. Bacteria were grown to an OD_600_ of 1.0, collected by centrifugation, then washed twice and resuspended in PBS. After addition of bacteria to each well, the plate was centrifuged at 1,700 g for 10 min to synchronize the infection. Three hours after the infection, LDH released from the dead cells was measured by the LDH cytotoxicity assay kit (Beyotime, Haimen, China). Cells treated with the cell lysis buffer provided by the kit were used as the control of 100% LDH release. The culture medium without cell was used to set the background LDH level. The cytotoxicity percentage was calculated following the manufacturer’s instruction.

### Western Blotting

Over night bacterial culture was diluted 1:100 in LB or 1:50 in LB with 5 mM EGTA (BBI Life Science, Shanghai, China) and incubated at 37°C with agitation. After 4 h, the supernatant of each culture was collected by centrifugation. Supernatants collected from equal numbers of bacteria were loaded to a 12% sodium dodecyl sulfate-polyacrylamide gel (SDS-PAGE). Then the proteins were transferred to a polyvinylidene difluoride (PVDF, Millipore, USA) membrane, and probed with a rabbit polyclonal antibody against ExoS (Li et al., 2013) at room temperature for 1 h. The membrane was washed three times with PBS containing 0.2% tween-20 (Tian Jin chemical reagent company, Tianjin, China), followed by incubation with a horseradish peroxidase-conjugated goat anti-rabbit IgG (Millipore, USA) at room temperature for 1 h. The signal was detected with the ECL-plus kit (Millipore, USA).

### Cell Culture and HL-60 Cell Differentiation

HL-60 cells (ATCC, USA) were cultured in RPMI 1640 medium (Hyclone, USA) with 10% (vol/vol) heat-inactivated fetal bovine serum (Gibco, Australia) and penicillin G (100 U/ml) and streptomycin (100 μg/ml; Hyclone, USA). The cells were cultured at 37°C with 5% CO_2_. Differentiation of the HL-60 cells was conducted as previously described (Chen and Seifert, 2011). Briefly, HL-60 cells were diluted to ∼4.5 × 10^5^ cells/ml and 1.3% dimethylsulfoxide (Sigma, USA) was added to the medium. The cells were then cultured for 6–7 days before use.

### Measurement of Reactive Oxygen Species (ROS) Levels

The ROS production levels were determined as previously described with slight modifications (Wu and Hsu, 2009). Briefly, differentiated HL-60 cells were washed once with warm Hank’s balanced salt solution (HBSS; Hyclone, USA) and diluted to 7.5 × 10^4^ cells/ml in HBSS containing 100 μM luminol (Sigma, USA) and 5 units per ml horseradish peroxidase (Sigma, USA). Two hundreds microliter cell suspension was added to each well of a 96-well plate, followed by incubation at 37°C for 10 min. Then the cells were infected with wild-type PAK or the Δ*eno* mutant at a multiplicity of infection (MOI) of 30. The ROS levels were measured every 3 min for 4 h with a Luminoskan Ascent Luminometer (Varioskan Flash, Thermo Scientific, USA).

### Growth Inhibitory Effect of Differentiated HL-60 Cells

Bacteria were grown to an OD_600_ of 1.0, collected by centrifugation and washed three times with sterile PBS. Then 1 × 10^7^ bacteria of each strain were incubated with 1 × 10^6^ undifferentiated or differentiated HL-60 cells in 200 μl RPMI 1640 medium at 37°C. At indicated time points, the live bacterial numbers were determined by serial dilution and plating. The growth inhibitory rate of each strain was calculated by dividing the live bacterial number incubated with differentiated HL-60 cells by the live bacterial number incubated with undifferentiated HL-60 cells.

### H_2_O_2_ Susceptibility Assay

Bacteria at an OD_600_ of 1.0 were collected and washed for three times with sterile PBS. Then the bacteria were diluted to 2 × 10^7^ CFU/ml in PBS and incubated with or without 10 mM H_2_O_2_ at 37°C for 15 min. The live bacterial numbers were determined by serial dilution and plating. The survival rate was calculated by dividing the live bacterial number with H_2_O_2_ treatment by the live bacterial number without H_2_O_2_ treatment.

### Ethical Statement

All animal experiments complied with Chinese national guidelines on the use of animals in research. The protocol was approved by the institutional animal care and use committee of the college of life sciences of Nankai University with a permit number: NK-04-2012.

## Results

### Enolase Is Required for *P. aeruginosa* Lung Colonization

**FIGURE 1:**
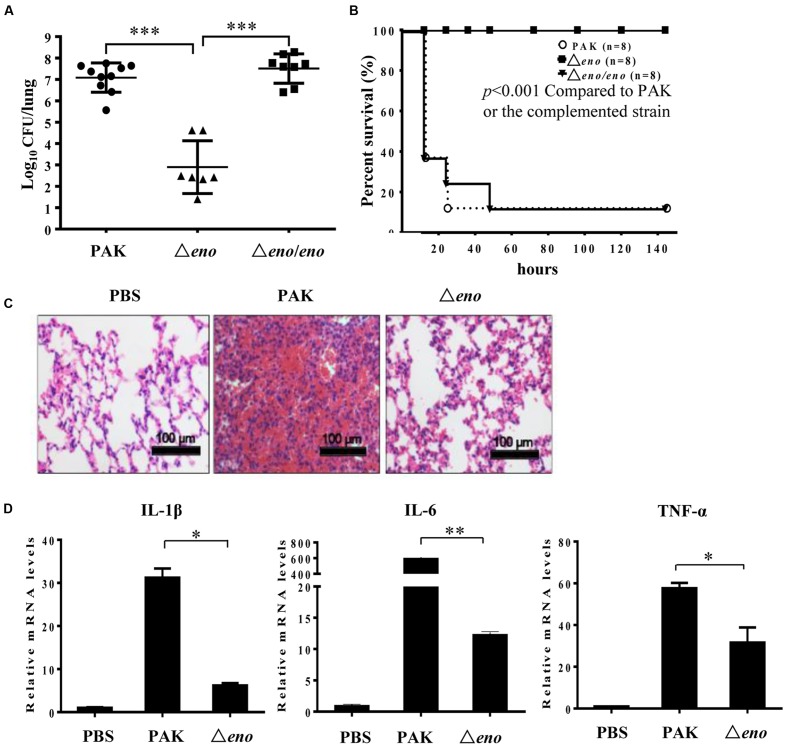
**Role of enolase in bacterial pathogenesis in a murine acute pneumonia model.** **(A)** Mice were infected with 2 × 10^7^ wild type PAK or the Δ*eno* mutant or the complemented strain intranasally. At 12 hpi, lungs from mice infected with no bacteria (PBS), or the indicated strains were recovered. Bacterial loads were determined by serial dilution and plating. ^∗∗∗^*P* < 0.001 by the Mann–Whitney test. **(B)** Each mouse was infected with 4 × 10^7^ bacteria intranasally. Survival of the mice was monitored at least twice per day for 6 days. The *P*-value was calculated by Kaplan–Meier survival analysis with a log rank test with the Prism software. **(C)** Each mouse was infected with 2 × 10^7^ bacteria intranasally. At 12 hpi, the lungs were fixed with paraformaldehyde (PFA), sectioned, and stained with hematoxylin and eosin. Images were taken with a 20× objective lens. **(D)** Total RNA was isolated from lungs of the infected mice. mRNA levels of IL1-β, IL-6, and TNF-α were determined by qRT-PCR. Bars represent means, and error bars represent standard deviation (SD). A representative of three independent experiments with similar results is shown. ^∗^*P* < 0.05; ^∗∗^*P* < 0.01 by student’s *t*-test.

To evaluate the role of enolase in *P. aeruginosa* pathogenesis, we utilized a murine acute pneumonia model as previously described (Sun et al., 2014). Six weeks old female BALB/c mice were infected intranasally with 2 × 10^7^ wild type PAK or an enolase deletion mutant (Δ*eno*). Twelve hours post-infection, lungs were isolated and homogenized. Bacterial loads were determined by serial dilution and plating. Compared to the wild type strain, the number of the Δ*eno* mutant was significantly lower (**Figure 1A**). For the complementation, an eno gene driven by its native promoter was cloned into pUC18T-mini-Tn7T-Gm and inserted into the chromosome (Choi and Schweizer, 2006). As shown in **Figure 1A**, complementation with an eno gene fully restored the bacterial load in the lung, indicating a role of enolase in bacterial growth in the lung. When, we grew the bacteria in LB, we noticed that the Δ*eno* mutant grows more slowly than wild type PAK (**Supplementary Figure S1A**). After 12 h *in vitro* growth, the bacterial number of the Δ*eno* mutant was ∼70% of that of the wild type PAK or the complemented strain (**Supplementary Figure S1B**). Given that there was ∼10^4^-fold difference in the bacterial load between wild type PAK and the Δ*eno* mutant infected mice, it is likely that factors other than slow growth contribute to the reduced bacterial number *in vivo*. To examine the role of enolase in virulence, we monitored the mortality rate in the acute pneumonia model. Infection with wild type PAK or the complemented strain resulted in 82.5% mortality rate, whereas no mouse died after infection with the Δ*eno* mutant (**Figure 1B**). Furthermore, lungs from mice at 12 hpi were subjected to H&E staining. Infection with wild type PAK resulted in severe occlusion with neutrophil infiltration, which was significantly milder in the Δ*eno* mutant infected lungs (**Figure 1C**). Consistently, lower mRNA levels of inflammatory cytokines, including IL-1β, IL-6, and TNF-α were detected in the lungs infected with the Δ*eno* mutant compared to those in the wild type PAK infected lungs (**Figure 1D**). Therefore, enolase is required for bacterial virulence in the acute pneumonia model.

### Mutation of *eno* Increases Bacterial Susceptibility to Oxidative Stresses

**FIGURE 2:**
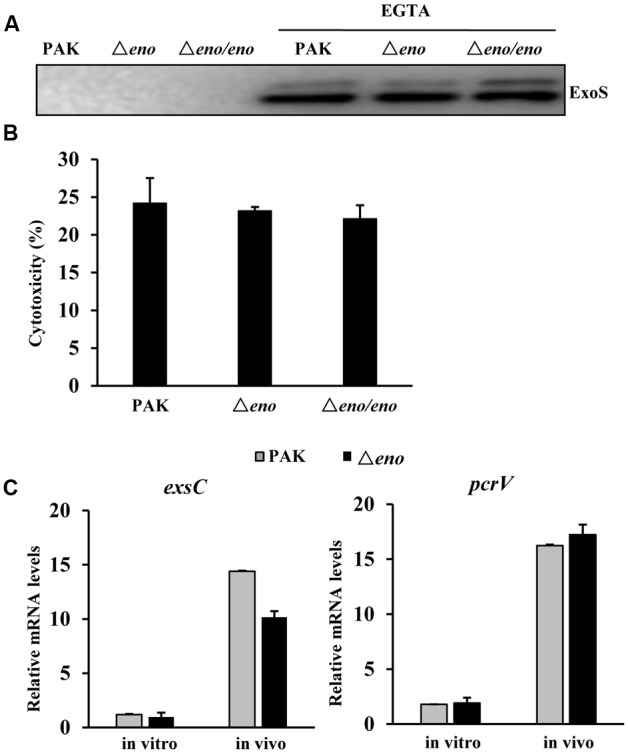
**Role of enolase in the regulation of T3SS.** **(A)** Wild type PAK, the Δ*eno* mutant and complemented strain were grown with or without 5 mM EGTA for 3 h. The supernatants from equal amount of bacteria were collected by centrifugation and the levels of ExoS were determined by western blot analysis. The data is representative of three independent experiments. **(B)** Cytotoxicity of wild type PAK, the Δ*eno* mutant and complemented strains to Hela cells. HeLa cells were infected with indicated strain at an MOI of 30 for 3 h. The relative cytotoxicity was determined by the LDH release assay. The error bars represente the standard errors. **(C)** Mice were infected with wild type PAK or the Δ*eno* mutant for 6 h. Bacteria were harvested from BALFs of the infected mice. Bacteria grown in LB were used as *in vitro* samples. RNAs were extracted from the bacteria and the relative levels of mRNA were determined by qRT-PCR. Results represent means ± SD.

In the mouse acute pneumonia model, neutrophils are rapidly recruited to the lung after infection and play a major role in the defense against bacteria (Wu et al., 2012; Ziltener et al., 2016). Induction and delivery of T3SS effector into neutrophils inhibit the bactericidal effects of those cells, enabling the bacterial colonization and dissemination (Diaz and Hauser, 2010; Howell et al., 2012; Rangel et al., 2015). Previously, we found that PNPase is required for the expression of the T3SS genes in the mouse acute pneumonia model (Chen et al., 2016). Since both enolase and PNPase are components of the RNA degradosome, they may share common regulatory targets. Thus, we examined the effect of *eno* mutation on the activity of the T3SS. Surprisingly, the expression and secretion of ExoS were similar between the Δ*eno* mutant and wild type PAK upon growth in calcium depleted LB medium, which is a typical *in vitro* T3SS inducing condition (**Figure 2A**), and the bacterial cytotoxicity were similar between wild type PAK and the Δ*eno* mutant (**Figure 2B**). We further examined the expression levels of T3SS genes during infection. Bacteria were isolated from bronchoalveolar lavage fluid (BALF) of infected mice. The mRNA levels of T3SS genes *exsC* and *pcrV* were determined by qRT-PCR with previously reported PA1805, PA1769, *rpsL*, and the 16S rRNA PA0668.1 as internal controls for normalization (Savli et al., 2003; Ruzin et al., 2007; Son et al., 2007; Sun et al., 2014). Similar mRNA fold of changes (within 1.2-fold difference) were observed between these internal controls. Therefore, we used the PA1805 as the internal control in this study. As shown in **Figure 2C**, the expression levels of *exsC* and *pcrV* were similar between wild type PAK and the Δ*eno* mutant. In combination, these results suggest that mutation of the *eno* does not affect the expression of T3SS genes.

**FIGURE 3:**
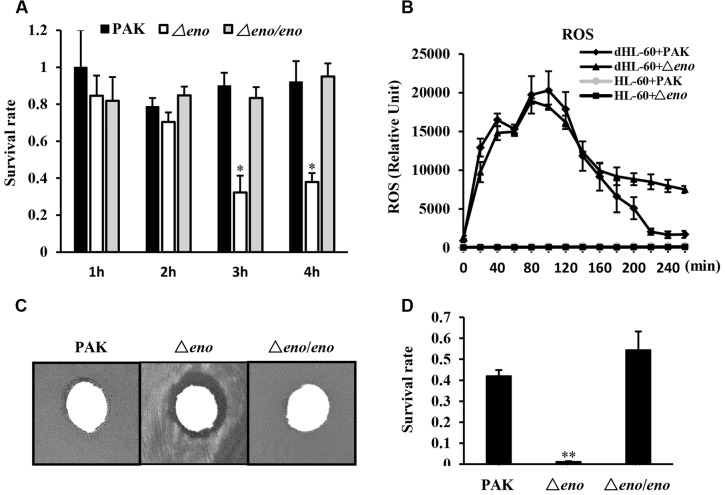
**Role of enolase in bacterial tolerance to oxidative stresses.** **(A)** Bacteria of indicated strains were incubated with differentiated HL-60 (dHL-60) cells or undifferentiated HL-60 cells. The live bacteria number was determined by plating. The relative growth rate of each strain was calculated as the ratio of live bacterial number in the presence of dHL-60 relative to that in the presence of undifferentiated HL-60. ^∗^*P* < 0.05 compared to PAK or the complemented strain by student’s *t*-test. **(B)** Differentiated or undifferentiated HL-60 cells were incubated with PAK or the Δ*eno* mutant. ROS levels were determined by a fluorescence spectrophotometer at different time points. **(C)** Circular filter paper was immersed in H_2_O_2_ (250 mM) for 5 s and placed in the center of plates that were spread with PAK, the Δ*eno* mutant or complemented strain. **(D)** Indicated strains were treated with H_2_O_2_ (10 mM) for 10 min and the numbers of live bacteria were determined by serial dilution and plating. ^∗∗^*P* < 0.01 compared to PAK or the complemented strain by student’s *t*-test.

Next, we compared the impact of neutrophils on the Δ*eno* mutant and wild type PAK. The bacteria were incubated with differentiated HL-60 (designated as dHL-60 hereafter) and undifferentiated HL-60 in RPMI-1640 medium. Compared to wild type PAK, the Δ*eno* mutant was more susceptible to the dHL-60 mediated growth inhibition (**Figure 3A**). A major bactericidal mechanism of neutrophils is production of ROS (Arai et al., 2001; Alalwani et al., 2009). As shown in **Figure 3B**, dHL-60 generated large amount of ROS upon encountering PAK or the Δ*eno* mutant. Therefore, we suspected that the Δ*eno* mutant is more susceptible to oxidative stresses. Indeed, in a disk diffusion assay, H_2_O_2_ caused bigger inhibition zone on the Δ*eno* mutant than that on the wild type PAK (**Figure 3C**). And treatment with H_2_O_2_ resulted in significant lower survival rate of the Δ*eno* mutant (**Figure 3D**). Complementation with an *eno* gene restored the bacteria tolerance to H_2_O_2_ (**Figure 3D**). These results suggest that enolase is involved in the bacterial tolerance to oxidative stresses.

### Mutation of *eno* Resulted in Down Regulation of *ahpB* and *ahpC*

**FIGURE 4:**
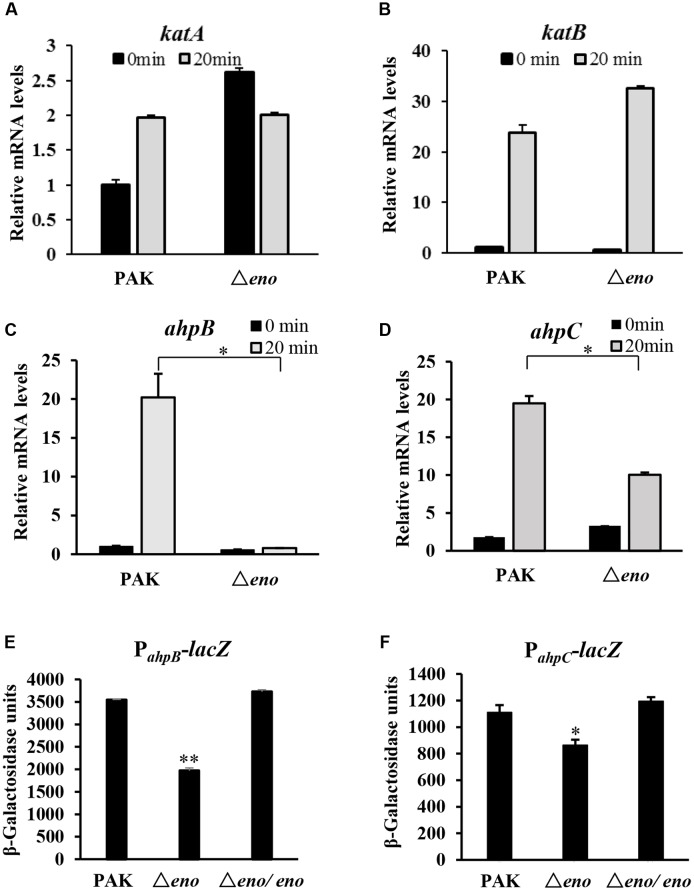
**Expression of oxidative stress response genes.** Wild type PAK and Δ*eno* mutant strain were treated with 10 mM H_2_O_2_ for 20 min, followed by RNA extraction. The relative mRNA levels of *katA* **(A)**, *katB* **(B)**, *ahpB* **(C)**, and *ahpC* **(D)** were determined by qRT-PCR. ^∗^*P* < 0.05 compared to the wild type PAK by Student’s *t*-test. Results represent means ± SD, and data are representative of three independent experiments. Indicated strains containing P*_ahpB_*- **(E)** or P*_ahpC_*-lacZ **(F)** transcriptional fusion were treated with 10 mM H_2_O_2_ for 30 min. The bacteria were collected, followed by β-Galactosidase assay. ^∗^*P* < 0.05, ^∗∗^*P* < 0.01 compared to the wild type PAK or the *eno* complement strain by Student’s *t*-test. Results represent means ± SD, and data are representative of three independent experiments.

In *P. aeruginosa*, the chromosomally encoded catalases (KatA and KatB), and alkyl hydroperoxide reductases (AhpB and AhpC) play important roles in the bacterial tolerance to oxidative stresses (Hassett et al., 1992; Ma et al., 1999). Thus, we examined whether enolase affects the expression of those genes. In wild type PAK, treatment with H_2_O_2_ induced the expression of *katA*, *katB*, *ahpB*, and *ahpC*. In the *eno* mutant, similar expression levels of *katA* and *katB* were observed (**Figures 4A,B**), except for the level of *katA* in the absence of H_2_O_2_, which was higher than that in wild type PAK (**Figure 4A**). However, the mRNA levels of *ahpB* and *ahpC* in the Δ*eno* mutant were 20- and 2-fold lower, respectively, in the presence of H_2_O_2_ (**Figures 4C,D**). To further confirm the expression levels of *ahpB* and *ahpC*, we constructed transcriptional fusions of *ahpB* promoter (P*_ahpB_*) or *ahpC* promoter (P*_ahpC_*) with *lacZ* reporter gene. In the presence of H_2_O_2_, the expression levels of *ahpB-lacZ* and *ahpC-lacZ* were reduced by ∼45 and 35% in the Δ*eno* mutant, respectively (**Figures 4E,F**).

In *P. aeruginosa*, OxyR activates the expression of *katA*, *katB*, *ahpB*, and *ahpC* in response to oxidative stresses (Heo et al., 2009). Since the expression levels of *katA* and *katB* were similar between wild type PAK and the Δ*eno* mutant in the presence of H_2_O_2_, we suspect that the expression and function of OxyR are normal in the *eno* mutant. Indeed, the mRNA levels of OxyR were similar between wild type PAK and the *eno* mutant with or without H_2_O_2_ treatment (**Supplementary Figure S2A**). In addition, expression of *prpL*, *toxA*, and *rgsA*, under the control of OxyR, was not affected by the mutation of *eno* (**Supplementary Figures S2B–D**). These results suggest that enolase affects the expression of *ahpB* and *ahpC* independent of the OxyR.

**FIGURE 5:**
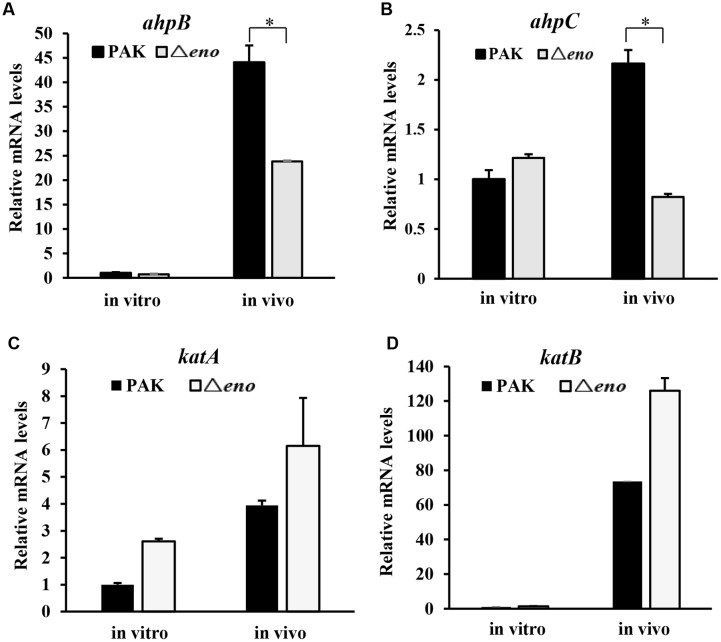
**Role of enolase in the expression of oxidative stress response genes *in vivo*.** Mice were infected with wild type PAK or the Δ*eno* mutant for 6 h. Bacteria were harvested from BALFs of the infected mice. Bacteria grown in LB were used as *in vitro* samples. RNA was extracted from the bacteria and the relative mRNA levels of *ahpB* **(A)**, *ahpC* **(B)**, *katA* **(C)**, and *katB* **(D)** were determined by qRT-PCR. Results represent means ± SD. ^∗^*P* < 0.05 compared to wild type PAK by student’s *t*-test.

Next, we examined the expression levels of *katA*, *katB*, *ahpB*, and *ahpC* in bacteria during mouse lung infection. At six hpi, bacteria were collected from BALF, followed by RNA extraction and qRT-PCR. The mRNA levels of *katA* and *katB* in the Δ*eno* mutant were slightly higher than those in wild type PAK (**Figures 5C,D**), however, the *ahpB* and *ahpC* mRNA levels were lower in the Δ*eno* mutant (**Figures 5A,B**).

### Overexpression of *ahpB* in the Δ*eno* Mutant Restores the Bacterial Tolerance to H_2_O_2_ and Virulence

**FIGURE 6:**
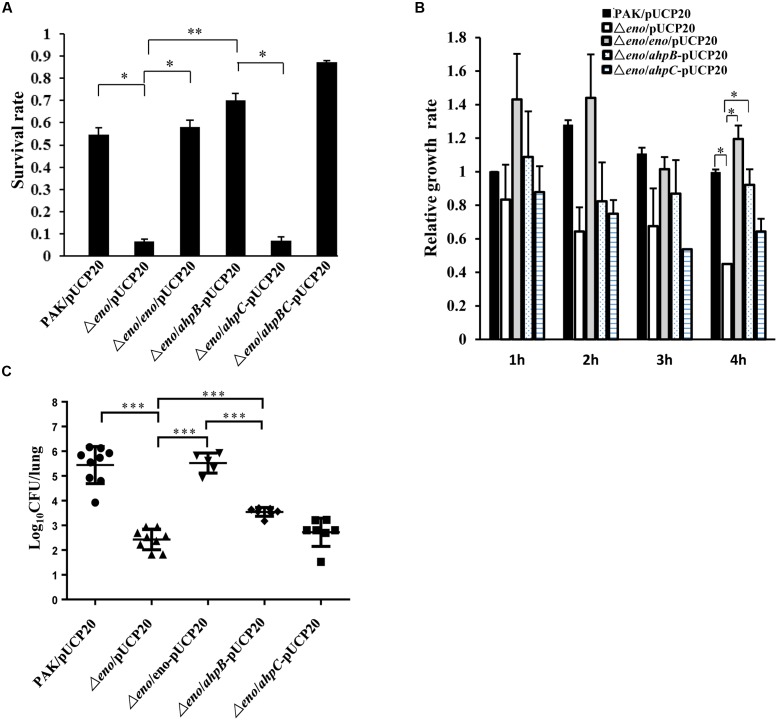
**Effect of overexpression of *ahpB* or *ahpC* on bacterial tolerance to H_2_O_2_ and virulence in the Δ*eno* mutant.** Empty vector pUCP20 was transferred into wild type PAK, the Δ*eno* mutant and the complemented strain, and plasmid overexpressing *ahpB*, *ahpC*, or both of the genes was transferred into the Δ*eno* mutant. **(A)** The indicated strains were treated with H_2_O_2_ (10 mM) for 10 min and the live bacteria numbers were determined by serial dilution and plating. Results represent means ± SD, and data are representative of three independent experiments. ^∗^*P* < 0.05, ^∗∗^*P* < 0.01 by Student’s *t*-test. **(B)** The bacteria were incubated with dHL-60 cells and undifferentiated HL-60 cells. The live bacteria number was determined by plating. The relative growth rate of each strain was calculated as the ratio of live bacterial number in the presence of dHL-60 relative to that in the presence of undifferentiated HL-60. **(C)** Mice were infected with the indicated strains intranasally. At 16 hpi, lungs from the infected mice were isolated. Bacterial loads were determined by serial dilution and plating. ^∗∗∗^*P* < 0.001 by the Mann–Whitney test.

The *in vitro* and *in vivo* results shown above demonstrate defective expression of *ahpB* and *ahpC* in the Δ*eno* mutant, which might be the cause of reduced tolerance to oxidative stresses. To test this further, we overexpressed the two genes individually or together in the Δ*eno* mutant and examined the bacterial survival rates after H_2_O_2_ treatment. As shown in **Figure 6A**, overexpression of *ahpB* but not *ahpC* in the Δ*eno* mutant restored the survival rate. Compared to *ahpB* alone, co-overexpression of *ahpB* and *ahpC* only slightly increased the bacterial survival rate. Consistently, overexpression of *ahpB* but not *ahpC* in the Δ*eno* mutant restored the bacterial growth in the presence of dHL60 (**Figure 6B**).

In the mouse acute pneumonia model, overexpression of *ahpB* in the Δ*eno* mutant increased the average bacterial load by ∼10-fold. However, overexpression of *ahpC* had no effect on the bacterial load (**Figure 6C**). In addition, overexpression of *ahpB* in the Δ*eno* mutant did not alter the bacterial growth rate in LB medium (**Supplementary Figure S1**). Therefore, these results suggest that down regulation of *ahpB* is the major cause of decreased tolerance to H_2_O_2_ and the reduced bacterial load of the Δ*eno* mutant.

## Discussion

In this study, we show that enolase is required for the virulence of *P. aeruginosa* in a murine acute pneumonia model. Further experimental results demonstrated that enolase affects the expression of two of the oxidative stress responsive genes, *ahpB* and *ahpC*. Mutation of *eno* abolished H_2_O_2_ induced expression of *ahpB*, but only partially affected the expression of *ahpC*. By overexpressing *ahpB* or *ahpC* in the Δ*eno* mutant, we demonstrate that *ahpB* plays a major role in the reduced bacterial tolerance to oxidative stresses and virulence.

OxyR plays a major role in the regulation of oxidative stress responsive genes (Wei et al., 2012; Jo et al., 2015). In the Δ*eno* mutant, the mRNA level of *oxyR* is similar to that in the wild type strain. Expression levels of known OxyR regulated genes, including *katA*, *katB*, toxA, *prpL*, *rgsA* are similar between the Δ*eno* mutant and wild type strain in the presence of H_2_O_2_. These results suggest that enolase is unlikely to affect the protein level and function of OxyR. We thus suspect that enolase might affect the expression of an unknown regulatory gene for the *ahpB* and/or *ahpC*. The N-terminus coding region of *ahpB* overlaps with PA0847, which is transcribed in the opposite direction. Thus, the promoters of *ahpB* and PA0847 should be inside the coding region of each other. We suspected that the transcription initiation or elongation of *ahpB* might be interfered by the RNA polymerase complex transcribing PA0847. However, the promoter activity of PA0847 in the Δ*eno* mutant was only 20% higher than that in the wild type strain in the presence of H_2_O_2_, as revealed by a β-galactosidase assay with a PA0847 promoter *lacZ* transcriptional fusion. Therefore, it is likely that other regulatory genes are involved in the regulation of *ahpB.*

Enolase belongs to a glycolytic enzyme, catalyzing the reversible dehydration of 2-phosphoglycerate to phosphoenolpyruvate (Sekowska et al., 2004). Besides, enolase also forms a complex with PNPase, a RNA helicase RhlB and ribonuclease E (RNase E), namely the RNA degradosome (Callaghan et al., 2004; Carpousis, 2007). The RNA degradosome plays an important role in RNA processing (Carpousis, 2007; Mildenhall et al., 2016). In *Salmonella enterica*, RNase E is involved in the regulation of genes required for intracellular replication (Yang et al., 2008), while in *Yersinia pseudotuberculosis*, RNase E regulates the expression of T3SS genes (Yang et al., 2008). PNPase plays important role in bacterial responses to various environmental stresses (Goverde et al., 1998; Clements et al., 2002; Len et al., 2004; Rosenzweig et al., 2005; Anderson and Dunman, 2009; Lawal et al., 2011). In *Yersinia*, it has been demonstrated that PNPase is required for the expression of T3SS and bacterial virulence (Rosenzweig et al., 2005). Our previous study in *P. aeruginosa* demonstrated that deletion of the RNA binding domains of PNPase leads to defective T3SS and attenuated virulence (Chen et al., 2016). However, the *in vitro* and *in vivo* experiments shown in this study demonstrated a normal T3SS function in the Δ*eno* mutant. These results suggest that enolase and other components of the RNA degradosome might affect the expression of distinctive subsets of genes.

In addition, enolase is a major type of moonlighting proteins, which are a group of proteins that have more than one unique biological functions (Henderson and Martin, 2011; Henderson, 2014). Enolase has been found on the cell surface of a growing number of bacteria and play roles in bacterial virulence (Henderson and Martin, 2011; Henderson, 2014). For example, the surface exposed enolase functions as an adhesion by binding to host plasminogen in various Streptococci, including *Streptococcus pneumoniae*, *S. pyogenes*, *S. gordonii*, *S. mutans*, *S. suis*, and *S. canis* (Pancholi and Fischetti, 1998; Ge et al., 2004; Esgleas et al., 2008; Kesimer et al., 2008; Fulde et al., 2013; Figueiredo et al., 2015). Mutation of the plasminogen bringing site of enolase reduced the bacterial virulence of *S. pneumoniae* in a murine intranasal infection model (Bergmann et al., 2003). Immunization with the enolase of *S. suis* conferred protection to mice against infection by the bacteria (Feng et al., 2009). In Gram negative bacteria, *Borrelia burgdorferi* and *Aeromonas hydrophila*, enolase has been found on cell surface (Carlson et al., 2007; Sierra et al., 2010). The plasminogen binding motif of enolase contributes to bacterial virulence of *A. hydrophila*, and immunization with enolase conferred protection (Sierra et al., 2010). Here in this study, we demonstrated that overexpression of *ahpB* in the Δ*eno* mutant fully restored the bacterial tolerance to H_2_O_2_, but only partially restored the virulence in the mouse pneumonia model. These results suggest the enolase might play additional roles in the virulence of *P. aeruginosa*. Further studies on the subcellular location and regulatory functions of the enolase are needed to fully elucidate the role of enolase in bacterial pathogenesis.

## Author Contributions

Conceived and designed the experiments: WW, YW, YJ, and SJ. Performed the experiments: YW, FC, YL, RC, CL, XP, and YJ. Analyzed the data: YW, QZ, WW, ZC, SJ, and YJ. Wrote the paper: YW, WW, and SJ.

## Conflict of Interest Statement

The authors declare that the research was conducted in the absence of any commercial or financial relationships that could be construed as a potential conflict of interest.

## Supplementary Material

FIGURE S1 **Growth of strains in LB medium. Same number of bacteria of each indicated strain were inoculated in LB and cultured at 37°C with agitation.** **(A)** Growth curve of indicated strain in LB. The OD_600_ of each culture was monitored every hour for 12 h. **(B)** After 12-h growth, the bacterial number of each strain was determined by serial dilution and plating.

FIGURE S2 **Expression of *oxyR* and genes regulated by it.** Wild type PAK and the Δ*eno* mutant were treated with 10 mM H_2_O_2_ for 20 min. Total RNA was isolated and the relative mRNA levels of *oxyR* **(A)**, *prpL* **(B)**, *toxA* **(C)**, and *rgsA* **(D)** were determined by qRT-PCR. Results represent means ± SD, and data are representative of three independent experiments.
