# Supplementary figures and images for: Pseudomonas aeruginosa Enolase Influences Bacterial Tolerance to Oxidative Stresses and Virulence
Source: Front Microbiol. 2016 Dec 15;7:1999. doi: 10.3389/fmicb.2016.01999 (PMC5156722; doi:10.3389/fmicb.2016.01999)

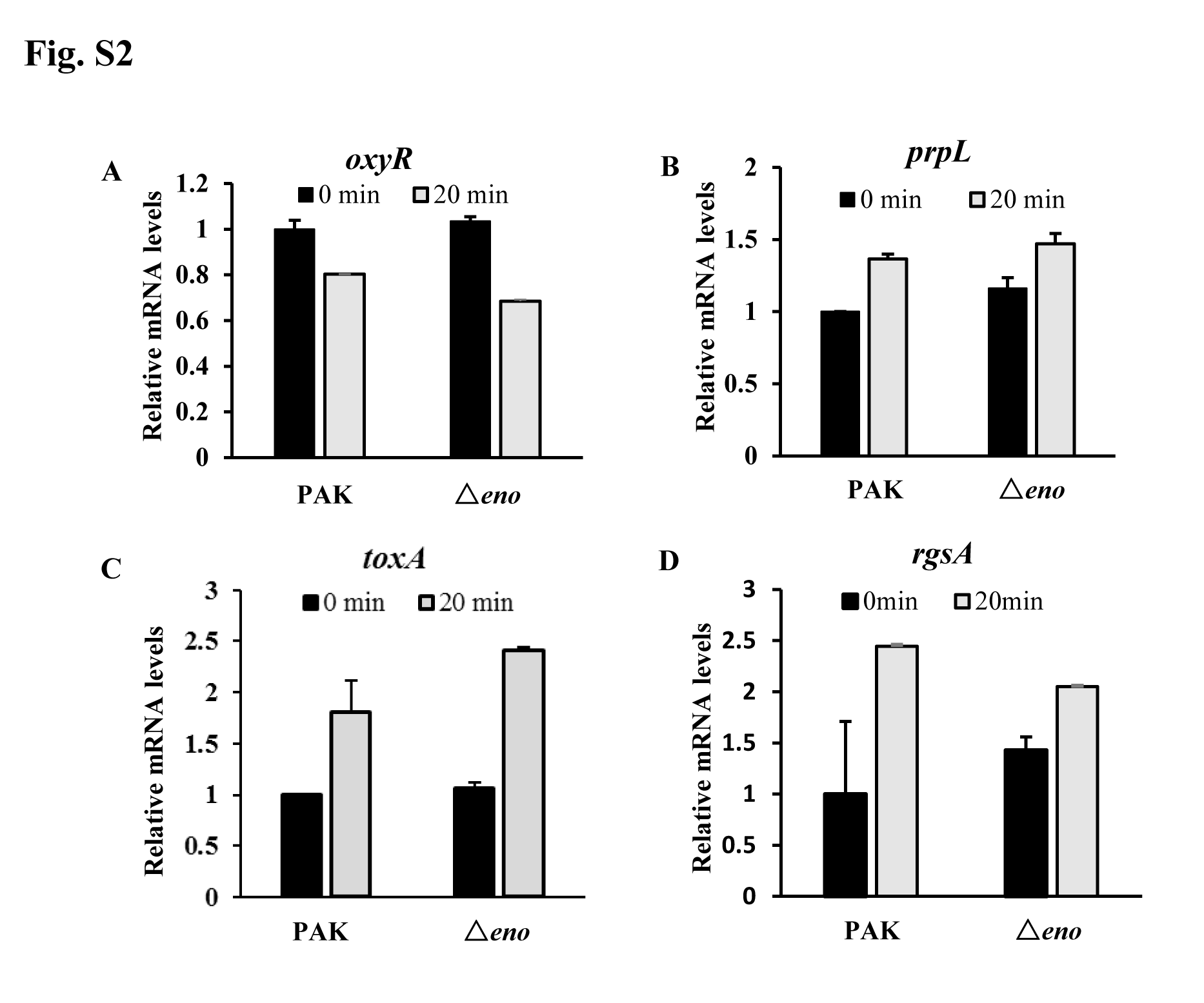

Supplement: FIGURE S1 — Growth of strains in LB medium. Same number of bacteria of each indicated strain were inoculated in LB and cultured at 37°C with agitation. (A) Growth curve of indicated strain in LB. The OD600 of each culture was monitored every hour for 12 h. (B) After 12-h growth, the bacterial number of each strain was determined by serial dilution and plating. [file Image_1.TIF]

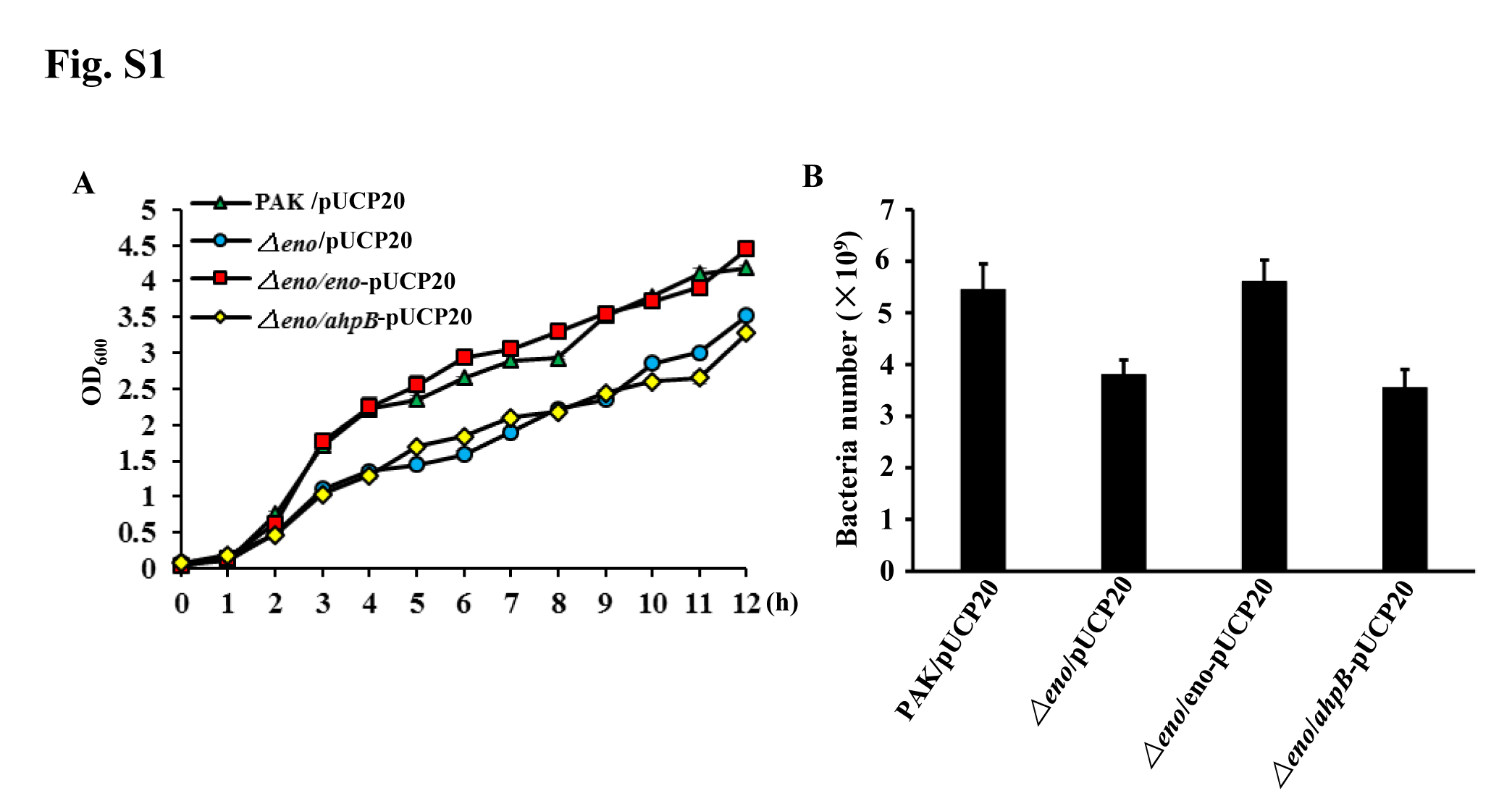

Supplement: FIGURE S2 — Expression of oxyR and genes regulated by it. Wild type PAK and the Δeno mutant were treated with 10 mM H2O2 for 20 min. Total RNA was isolated and the relative mRNA levels of oxyR (A), prpL (B), toxA (C), and rgsA (D) were determined by qRT-PCR. Results represent means ± SD, and data are representative of three independent experiments. [file Image_2.TIF]
