# Supplementary material for: Pseudomonas aeruginosa Enolase Influences Bacterial Tolerance to Oxidative Stresses and Virulence
Source: Front Microbiol. 2016 Dec 15;7:1999. doi: 10.3389/fmicb.2016.01999 (PMC5156722; doi:10.3389/fmicb.2016.01999)
Supplement: Supplementary file 3 [file Table_1.PDF]

Table S1. Primers used in this study.

**Primers**	**Nucleotide sequence (5′-3′)**
**Cloning of upstream and downstream fragments for *eno* deletion**	
eno-up-F	TACTCAGAATTCGCACGACTGCTATGCCAAGGAC（EcoRI）
eno-up-R	TACTCAGGATCCTCACGTCCGCTTCAACGGTAGG（BamHI）
eno-down-F	TACTCAGGATCCGCCTAGTCTGGTACGGTGAAGG（BamHI）
eno-down-R	TACTCAAAGCTTGGAACATCTGCGGCGTATAGGC（HindIII）
**Cloning of *eno* coding region for complementation**	
C-p-*eno*-F	TACTCAAAGCTTCGCTCATCCATTGCTTCTTCCC（HindIII）
C-p-*eno*-R	TACTCAGGATCCCAATGAAGAAACAACACCACCC（BamHI）
C-*eno*-F	TACTCAGGATCCTCTCCTCAGACCGCCCCGTTCA（BamHI）
C-*eno*-R	TACTCAGAGCTCTCAGCCGCGGAATTCCGCGCGA（SacI）
**Construction of *ahpB* and *ahpC* overexpression plasmids**	
Exp-*ahpB*-F	TACTCAGAATTCATGAGCGTACTGGTCAACAAGCA (EcoRI)
Exp-*ahpB*-R	TACTCAGGATCCTCACAGTGCCTCGGCGTTC (BamHI)
Exp-*ahpC*-F	TACTCAGGATCCATGTCCCTGATCAACACTCAAG (BamHI)
Exp-*ahpC*-R	TACTCAAAGCTTTTAGATCTTGCCGACCAGGT（HindIII）
**Construction of *ahpB*- and *ahpC-lacZ* transcriptional fusions**	
p-*ahpB*-F	TACTCAGAATTCGCTTCAACTCGAAGTCCAG (EcoRI)
p-*ahpB*-R	TACTCAGGATCCGATGGGTGAACTGCGAGTC (BamHI)

p-*ahpC*-F	TACTCA<u>GAATTC</u>GACCATCCTGGTGCTGGTC (EcoRI)
p-*ahpC*-R	TACTCA<u>GGATCC</u>TGCCCTTCAGGGATTCCTC (BamHI)
**qRT-PCR primers**	
q-*ahpB*-F	CCTTGCGTGCTTCGTTCC
q-*ahpB*-R	AGACCTCGCCGTGTTCCTC
q-*ahpC*-F	GCAAGTGGTCGGTCCTGAT
q-*ahpC*-R	AGAAGTGGGTGTCGGTGGT
q-*katA*-F	GCGGCTACCTATCGCTACAA
q-*katA*-R	TCCGGCGAGAAACCGATAC
q-*katB*-F	GAAACAGGTGGCTGAAGTCC
q-*katB*-R	CGACCTGTTCGGTTTCCTG
q-*oxyR*-F	GTTCCCGCACCTGATTCCC
q-*oxyR*-R	CAGGAGCAGGCTCTTGTCGTT
q-*prpL*-F	TATCGTATTTCGCCGACTCCC
q-*prpL*-R	GCGAGTTGCCGTTGTTCAG
q-*rgsA*-F	CCGCAAGGGTATTCCAAA
q-*rgsA*-R	AAGGTACGCATTCCTCGGT
q-toxA-F	CGAGATGGGCGACGAGTTG
q-*toxA*-R	TGATGACCGTGGGCTTGATGT

The underlined sequence represents endonuclease recognition site.

F: forward ; R：reverse
